# Technique, protocols and adverse reactions for contrast-enhanced spectral mammography (CESM): a systematic review

**DOI:** 10.1186/s13244-019-0756-0

**Published:** 2019-08-02

**Authors:** Moreno Zanardo, Andrea Cozzi, Rubina Manuela Trimboli, Olgerta Labaj, Caterina Beatrice Monti, Simone Schiaffino, Luca Alessandro Carbonaro, Francesco Sardanelli

**Affiliations:** 1 Department of Biomedical Sciences for Health, Università degli Studi di Milano, Via Mangiagalli 31, 20133 Milan, Italy; 2 Department of Morphology, Surgery and Experimental Medicine, Section of Radiology, University of Ferrara, Via Ludovico Ariosto 35, 44121 Ferrara, Italy; 3 Unit of Radiology, IRCCS Policlinico San Donato, Via Morandi 30, 20097 San Donato Milanese, Italy

**Keywords:** Breast, Contrast media, Drug-related side effects and adverse reactions, Mammography, Radiation dosage

## Abstract

We reviewed technical parameters, acquisition protocols and adverse reactions (ARs) for contrast-enhanced spectral mammography (CESM). A systematic search in databases, including MEDLINE/EMBASE, was performed to extract publication year, country of origin, study design; patients; mammography unit/vendor, radiation dose, low-/high-energy tube voltage; contrast molecule, concentration and dose; injection modality, ARs and acquisition delay; order of views; examination time. Of 120 retrieved articles, 84 were included from 22 countries (September 2003–January 2019), totalling 14012 patients. Design was prospective in 44/84 studies (52%); in 70/84 articles (83%), a General Electric unit with factory-set kVp was used. Per-view average glandular dose, reported in 12/84 studies (14%), ranged 0.43–2.65 mGy. Contrast type/concentration was reported in 79/84 studies (94%), with Iohexol 350 mgI/mL mostly used (25/79, 32%), dose and flow rate in 72/84 (86%), with 1.5 mL/kg dose at 3 mL/s in 62/72 studies (86%). Injection was described in 69/84 articles (82%), automated in 59/69 (85%), manual in 10/69 (15%) and flush in 35/84 (42%), with 10–30 mL dose in 19/35 (54%). An examination time < 10 min was reported in 65/84 studies (77%), 120 s acquisition delay in 65/84 (77%) and order of views in 42/84 (50%) studies, beginning with the craniocaudal view of the non-suspected breast in 7/42 (17%). Thirty ARs were reported by 14/84 (17%) studies (26 mild, 3 moderate, 1 severe non-fatal) with a pooled rate of 0.82% (fixed-effect model). Only half of CESM studies were prospective; factory-set kVp, contrast 1.5 mL/kg at 3 mL/s and 120 s acquisition delay were mostly used; only 1 severe AR was reported. CESM protocol standardisation is advisable.

## Key points

- Eighty-four articles on CESM totalling 14012 patients were reviewed
- A 1.5 mL/kg contrast dose automatically injected at 3 mL/s was generally adopted
- Per-view average glandular dose ranged from 0.43 to 2.65 mGy
- Studies for contrast agent dose-finding and view acquisition ordering are lacking
- Adverse reaction rate (only one severe) was similar to that reported for CT

## Background

During the 1960s and 1970s, randomised controlled trials proved that screen-film mammography for breast cancer screening yields a reduction in breast cancer mortality [1]. Since the early 2000s, screen-film mammography was progressively replaced by digital mammography (DM), which improved performance especially in women under 50 years of age and in case of dense breasts, even though providing an intrinsically inferior spatial resolution [2]. In the last two decades, digital breast tomosynthesis brought substantial further improvements [3, 4], increasing cancer detection rate and reducing the recall rate [5].

Contrast-enhanced mammography is the combination of X-ray mammography with intravenous administration of iodinated contrast agent (ICA) [6]. It was first attempted using a digital subtraction technique [7–9], but this approach was soon abandoned due to difficulties in co-registration of unenhanced and contrast-enhanced images [10, 11]. In the last two decades, contrast-enhanced spectral mammography (CESM) has been introduced, based on dual-energy breast exposure (about 26–33 kVp and 44–50 kVp) after contrast administration, so that the pre-contrast exposure was no longer needed [10, 12]. CESM allows for the visualisation of enhancing findings over the normal unenhancing breast tissue, exploiting the increased contrast uptake of malignancies [6, 10, 13].

Original studies have investigated the use of CESM in a number of settings, such as evaluation of symptomatic women [14–17], screening recalls [18–22], local staging [23–32], pre- and post-operative evaluations [23, 24, 33–36] and neoadjuvant chemotherapy response monitoring [37–40]. In 2016, a first meta-analysis on CESM described a high pooled sensitivity (98%) albeit with a relatively low specificity (58%) [41], the latter partly caused by inexperience. A more recent meta-analysis [42] reported globally satisfying data for CESM-pooled sensitivity (89%) and specificity (84%), proposing it as an alternative to contrast-enhanced magnetic resonance imaging (MRI) and even suggesting CESM as a “useful triage test for initial breast lesions assessment” [41].

A time delay between the first appearance of new imaging techniques and their implementation in diagnostic routine is expected for many reasons, including not only the definition of indications but also the reproducibility of results. The latter is strongly influenced by technique details, such as contrast agent concentration, dose and injection rate, breast compression and positioning, exposure parameters and acquisition protocol. Indeed, the fact that CESM is variably performed across different centres, without an agreed and standardised technique, does not come as a surprise: this circumstance echoes the one observed for contrast-enhanced breast MRI in the 1990s, now settled by the publication of detailed international guidelines [43–46].

Therefore, the aim of this work was to review CESM studies focusing on adopted technique, contrast agent issues and acquisition workflow. This effort is crucial for future CESM investigations to be reproducible and comparable.

## Methods

### Study protocol

No ethics committee approval was needed for this systematic review. The study protocol was registered on PROSPERO (protocol CRD42018118554), the international prospective register of systematic reviews [47]. This systematic review was reported according to the Preferred Reporting Items for Systematic Reviews and Meta-Analyses (PRISMA) statement [48].

### Search strategy and eligibility criteria

In February 2019, a systematic search was performed on MEDLINE (PubMed, https://www.ncbi.nlm.nih.gov/pubmed/), EMBASE (Elsevier), the Cochrane Library (Cochrane Database of Systematic Reviews) and the Cochrane Central Register of Controlled Trials for articles that reported or may have reported CESM technique. A controlled vocabulary (medical subject headings in PubMed and EMBASE thesaurus keywords in EMBASE) was used. The search string was (cesm OR ‘contrast enhanced spectral mammography’/exp. OR ‘dual energy mammography’ OR ‘contrast enhanced digital mammography’/exp. OR ‘contrast-enhanced mammography’ OR ‘dual-energy subtraction mammography’ OR cedm OR cedsm OR ‘contrast enhanced spectral imaging’ OR ‘high energy and low energy digital mammography’) AND (‘procedures’/exp. OR ‘method’ OR ‘methods’ OR ‘procedure’ OR ‘procedures’ OR ‘technique’ OR ‘acquisition’/exp. OR ‘contrast medium’/exp. OR ‘contrast agent’ OR ‘contrast dye’ OR ‘contrast material’ OR ‘contrast media’ OR ‘contrast medium’ OR ‘radiocontrast medium’ OR ‘radiography contrast medium’ OR ‘roentgen contrast medium’ OR ‘image processing’/exp. OR ‘image processing’ OR ‘image processing, computer-assisted’ OR ‘processing, image’).

The search was limited to original studies on humans published in English, French and Spanish on peer-reviewed journals, with an available abstract. No publication date limits were applied. First article screening was performed by two independent readers (A.C. and M.Z., with 1- and 3-year experience in breast imaging, respectively) considering only title and abstract. Eligible articles were those that reported in the title or in the abstract the use of CESM technique or that could have contained these data in the manuscript. After downloading eligible articles, the full text was read for a complete assessment. Finally, references of included articles were hand-searched to check for further eligible studies.

### Data extraction

Data extraction was performed independently by the same two readers who performed the literature search. Disagreements were settled by consensus. For each analysed article, year of publication, institution (such as hospitals, imaging facilities, breast units including radiology sections or any other type of centre in which CESM is performed) and country origin as well as research groups, design, number of patients and demographics were retrieved. Mammography unit, vendor, radiation dose and technical features such as low- and high-energy peak kilovoltage (kVp), anode/filter combinations and exposure parameters were also extracted. Moreover, contrast agent type, dose and concentration were retrieved, as well as injection modality, if manual or automated, flow rate and additional post-contrast saline flush or “bolus chaser” if present. Furthermore, mild, moderate or severe adverse reactions to ICAs were extracted alongside strategies for their prevention. Regarding the acquisition protocol, time between contrast injection and first image acquisition and maximum examination duration were extracted. Regarding the order of views, we reported the acquisition sequence of the standard mammographic projections considering the craniocaudal (CC) and the mediolateral oblique (MLO) views, including the first side acquired. Missing data were requested to authors.

### Evidence synthesis

To avoid risk of data duplication bias, in case of articles published by the same research group, we considered the possibility of performing subgroup analysis: therefore, before delving into further analysis of protocol description, we chose to change our viewpoint from the number of articles reporting a specific protocol to the minimum number of times a protocol was reported by a single research group.

Regarding the pooled rate of adverse reactions related to ICA administration across studies, statistical analysis was performed using Comprehensive Meta-Analysis v2.2.057 (Biostat, Englewood, NJ, USA) using the meta-analysis model “Number of events and study population”. *I*^2^ statistics was first calculated to assess heterogeneity and the fixed-effect model was used to provide the rate of adverse reactions and 95% of confidence intervals (CI). The risk of publication bias was assessed by visually inspecting funnel plot and performing the Egger test [49].

## Results

### Studies

**Fig. 1:**
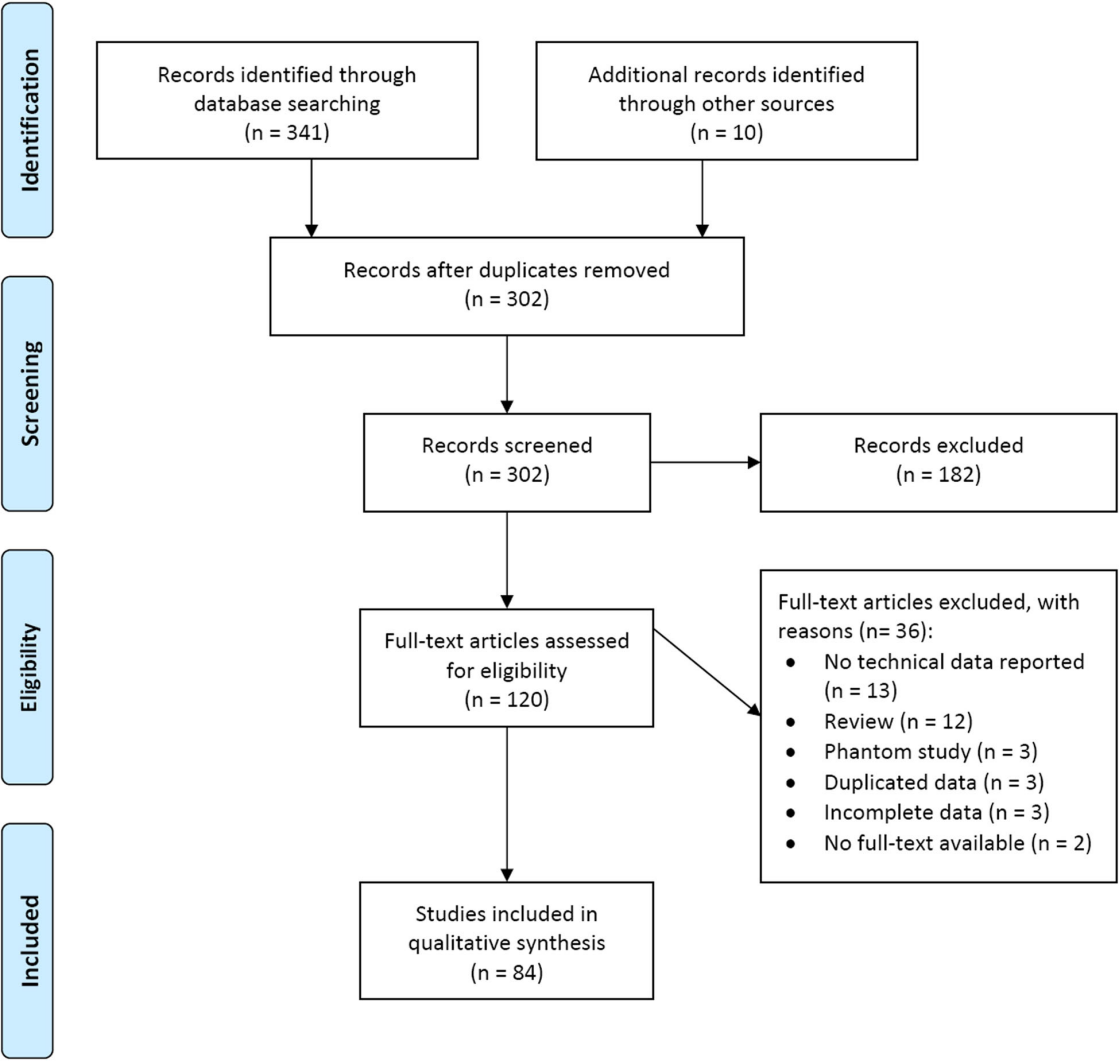
Flowchart of the study selection and exclusion for articles on contrast-enhanced spectral mammography

**Fig. 2:**
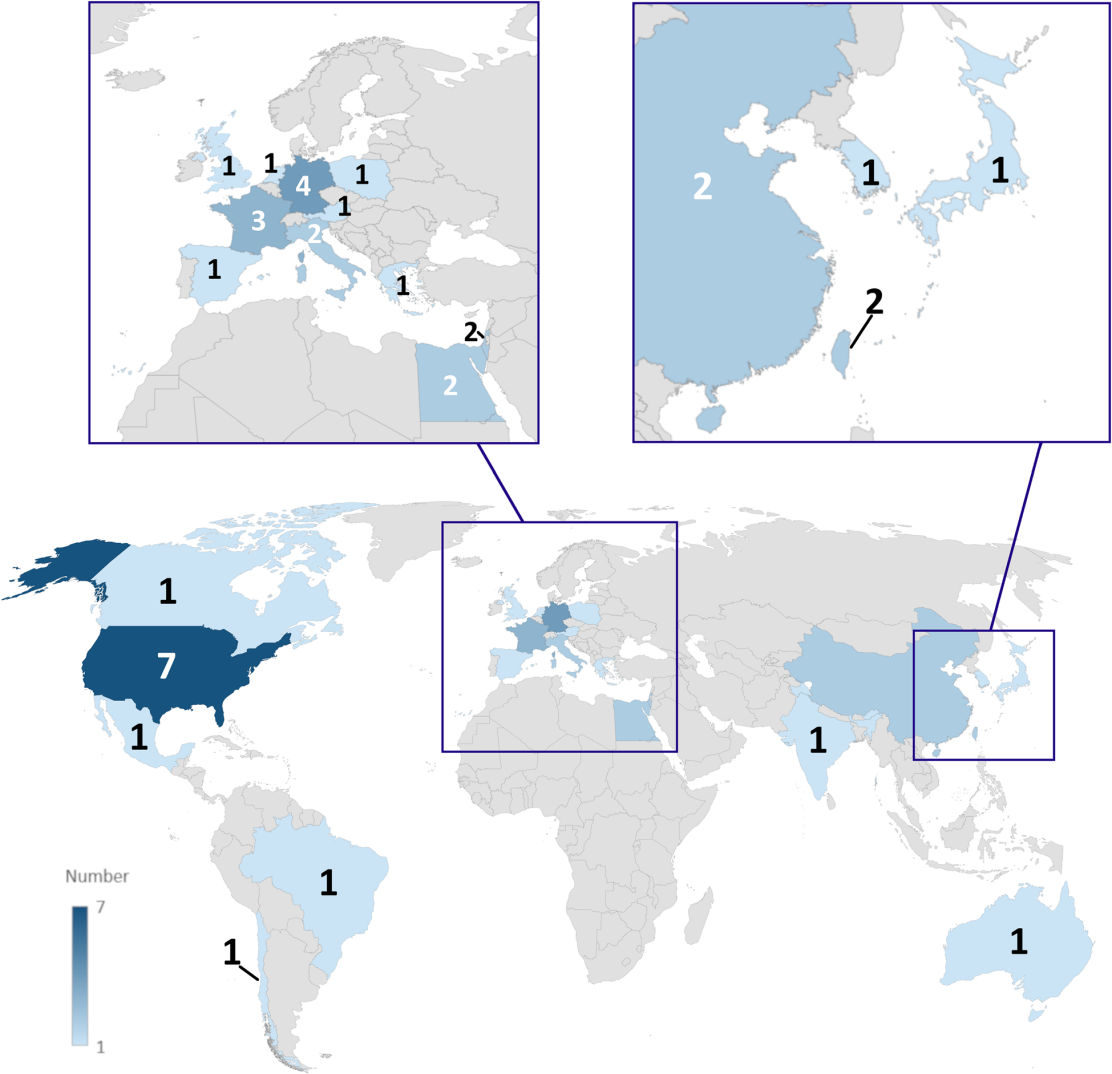
Geographic distribution of research groups which published results of clinical applications of contrast-enhanced spectral mammography. From very light blue to dark blue, the number of groups progressively increases from 1 to 7; grey colour means no publications

A flowchart of study selection is shown in Fig. 1. Of 120 retrieved articles, 84 (70%), published between September 2003 and January 2019, were analysed [7–10, 13–40, 50–101]; 40/84 (48%) being retrospective and 44/84 (52%) prospective (43/44 monocentric (98%) and 1/44 multicentric (2%); 54/84 (64%) articles investigated CESM diagnostic performance, whereas 30/84 (36%) focused on technical features. The geographic distribution of research groups is depicted in Fig. 2.

### Populations and settings

Data synthesis is reported in Table 1. The number of patients ranged from 5 [63] to 2303 [13], for a total of 14,012 patients, with mean or median age ranging from 45 years [40] to 66 years [23]. In 29/84 studies (35%), CESM was performed on patients from comprehensive databases of heterogeneous settings, such as pre- or post-operative evaluation, adjuvant or neoadjuvant chemotherapy response monitoring and equivocal findings at conventional imaging. The remaining 55 studies (65%) were individually centred on a unique setting. Twenty-seven studies (32%) performed CESM on suspicious cases from conventional imaging and screening recalls, 11 studies (13%) in a first-line screening setting, 7 (8%) performed CESM exclusively for known cancer staging, 4 (5%) in a pre-operative setting, 4 (5%) to assess and monitor the response to adjuvant chemotherapy and 2 (2%) in a post-operative setting.

**Table 1:** Main characteristics of the 84 analysed studies

Author/year	Ref.	Study design	Country of research group	Number of patients	Mean or median age (years)	Contrast agent type	Concentration (mgI/mL)	Dose (mL/kg)	Flow rate (mL/s)	Delay after injection (s)	Total exam time
Houben 2019	[22]	R	The Netherlands	147	61	Iopromide	300	1.5	3	120	
Barra 2018	[40]	P mono	Brazil	33	45	Iohexol	300	1.5	3	120	B
Bicchierai 2018	[93]	R	Italy	40	50	Iopromide	370	1.5	3	120	B
Danala 2018	[69]	R	USA	111		Iohexol	350	1.5	3	120	B
Deng 2018	[78]	R	Taiwan	141	48	Iohexol	350	1.5	3	120	B
Helal 2018	[25]	P mono	Egypt	300	54	Iohexol	300	1.5	3	120	B
Kim 2018	[87]	P mono	South Korea	84	51	Iohexol	350	1.5	2	120	B
Klang 2018	[88]	R	Israel	953	51	Iopamidol	370	1.5	3	120	B
Łuczyńska 2018	[36]	R	Poland	82	57	Iopromide	370	1.5	3	120	B
Moustafa 2018	[17]	P mono	Egypt	160		Iohexol	300	1.5	3	120	B
Navarro 2018	[90]	P mono	Chile	465	53	Ioversol	320	1.5			B
Patel 2018 (01)	[38]	P mono	USA	65	53	Iohexol	350	1.5	3	120	A
Patel 2018 (02)	[34]	R	USA	50	57	Iohexol	350	1.5	3	120	B
Patel 2018 (03)	[23]	R	USA	30	66	Iohexol	350	1.5	3	120	B
Phillips 2018	[82]	R	USA	45	53	Iohexol	350	1.5	3	120	
Sorin 2018	[92]	R	Israel	611	54	Iopamidol	370	1.5	3	120	B
Tohamey 2018	[51]	P mono	Egypt	178	46	Iohexol	300	1.5	3	120	B
Travieso-Aja 2018	[24]	R	Spain	158	51			1.5	3	120	B
Xing 2018	[84]	P mono	China	235	51	Iohexol	350	1.5	3	120	B
Barra 2017	[39]	R	Brazil	11	46	Iohexol	300	1–2	3	120	B
Bhimani 2017	[13]	R	USA	2303		Iopamidol	370	1.5	2	120	B
Fallenberg 2017	[76]	P multi	Germany	155	53	Iobitridol	300	1.5	3	120	A
Gluskin 2017	[63]	R	USA	5	59	Iohexol	350	1.5	3	150–180	A
Helal 2017 (01)	[28]	P mono	Egypt	98	50	Iohexol	300	1.5	3	120	B
Helal 2017 (02)	[99]	P mono	Egypt	30	47	Iohexol	300	1.5		120	
Houben 2017	[58]	R	The Netherlands	839	60	Iopromide	300	1.5	3	120	
Iotti 2017	[37]	P mono	Italy	54	54	Ioversol	350	1.5		120	
James 2017	[56]	R	USA	173		Iohexol	350	1.5	3	120	A
Jochelson 2017	[54]	P mono	USA	309	51	Iohexol	350	1.5	3	150–180	B
Knogler 2017	[94]	P mono	Austria	11	58	Iomeprol	400	2	3.5	90	
Lee-Felker 2017	[26]	R	USA	52	50	Iohexol	350		3	120	B
Lewis 2017	[16]	R	USA	208		Iohexol	350	1.5	3	120	B
Li 2017	[100]	R	USA	48	56	Iopamidol	370	1.5	1.5–2		B
Mori 2017	[74]	P mono	Japan	72	48	Iohexol	300	1.5	3	120	
Patel 2017 (01)	[27]	R	USA	88	62	Iohexol	350	1.5	3	120	B
Patel 2017 (02)	[65]	R	USA	410		Iohexol	350	1.5	3	120	B
Phillips 2017	[70]	P mono	USA	38	53	Iohexol	350	1.5	3	120	B
Richter 2017	[62]	R	Germany	118	58	Iopromide	300	1.5	2–3	120	
Saraya 2017	[18]	P mono	Egypt	34	54	Iohexol	300	1.5	4		C
Savaridas 2017	[75]	P mono	Australia	66	54			1.5	3	120	B
Sogani 2017	[80]	R	USA	278	51	Iohexol	350	1.5	3	150	A
Ali-Mucheru 2016	[33]	R	USA	351	62	Iohexol	350	1.5	3	120	B
Ambicka 2016	[29]	R	Poland	82	57	Iopromide	370	1.5	3	120	B
Brandan 2016	[77]	P mono	Mexico	18	51	Ioversol	300		4	60	B
Cheung 2016 (01)	[72]	R	Taiwan	256	48	Iohexol	350	1.5	3	120	A
Cheung 2016 (02)	[98]	R	Taiwan	87	54	Iohexol	350	1.5	3	120	B
Kamal 2016	[95]	R	Egypt	239	48	Iohexol	300	1.5	3	120	B
Kariyappa 2016	[68]	P mono	India	44		Iomeprol	350	1.5	3	120	B
Knogler 2016	[83]	P mono	Austria	15	58	Iomeprol	400	2	3.5	60–90	
Lalji 2016	[21]	R	The Netherlands	199	58	Iopromide	300	1.5	3	120	
Łuczyńska 2016 (01)	[50]	P mono	Poland	116	55	Iopromide	370	1.5	3	120	B
Łuczyńska 2016 (02)	[67]	P mono	Poland	193	55	Iopromide	370	1.5	3	120	B
Tardivel 2016	[19]	R	France	195	56	Iobitridol	300	1.5	3	120	B
Tennant 2016	[15]	R	UK	99	49						
Tsigginou 2016	[89]	P mono	Greece	216	55	Iopromide	300	1.5	2–3	120	B
Wang 2016	[97]	P mono	China	68	53	Iohexol	350	1.5	3	120	A
Yagil 2016	[71]	R	Israel	200	51	Iopamidol	370	1.5	3	120	B
Chou 2015	[14]	P mono	Taiwan	185	51	Iohexol	300	1.5	2	120	B
Elsaid 2015	[73]	P mono	Egypt	34	55	Iohexol	300	1.5	3		B
Hobbs 2015	[81]	P mono	Australia	49	55	Iohexol	350	1.5	3	120	B
Kamal 2015	[79]	R	Egypt	168		Iohexol	300	1.5	3	120	B
Lobbes 2015	[30]	R	The Netherlands	87	62	Iopromide	300	1.5	3	120	
Łuczyńska 2015 (01)	[91]	P mono	Poland	174	56	Iopromide	370	1.5	3	120	B
Łuczyńska 2015 (02)	[53]	P mono	Poland	102		Iopromide	370	1.5	3	120	
Badr 2014	[101]	P mono	France	75	54	Iohexol	300	1.5		120	B
Blum 2014	[31]	P mono	Germany	20	57	Iopamidol	300	1.5	3	120	
Cheung 2014	[86]	R	Taiwan	89	48	Iohexol	350	1.5	3	120–180	B
Fallenberg 2014 (01)	[85]	P mono	Germany	118	53	Iobitridol	300	1.5	3	120	B
Fallenberg 2014 (02)	[32]	P mono	Germany	80	54	Iobitridol	300	1.5	3	120	B
Francescone 2014	[66]	R	USA	88	50						
Jeukens 2014	[60]	R	The Netherlands	47	58	Iopromide	300	1.5	3	120	
Lobbes 2014	[20]	R	The Netherlands	113	57	Iopromide	300	1.5	3	120	
Łuczyńska 2014	[35]	P mono	Poland	152	56	Iopromide	370	1.5	3	120	B
Mokhtar 2014	[57]	P mono	Egypt	60		Iohexol	300	1.5		120	A
Travieso-Aja 2014	[64]	R	Spain	136	49			1.5	3	120	B
Hill 2013	[10]	R	Canada	98	57	Iobitridol	300	1.5	3	120	B
Jochelson 2013	[55]	P mono	USA	82	50	Iohexol	350	1.5	3	150–300	B
Dromain 2012	[52]	P mono	France	110	57	Iobitridol	300	1.5	3	120	A
Diekmann 2011	[61]	P mono	Germany	70	55	Iopromide	370	1	4	60/120/180	A
Dromain 2011	[59]	P mono	France	120	56	Iobitridol	300	1.5	3	120	A
Dromain 2006	[9]	P mono	France	20	63	Iohexol	300		3	30	B
Diekmann 2005	[8]	P mono	Germany	21		Iopromide	370	1	4	60/120/180	A
Jong 2003	[7]	P mono	Canada	22		Iohexol	300			60	B
Lewin 2003	[96]	P mono	USA	26	51	Iohexol	350		4–5	150	

*R* retrospective, *P mono* prospective monocentric, *P multi* prospective multicentric*, A* = total exam time < 5 min, *B* = total exam time between 5 and 10 min, *C* = total exam time > 10 min

Timing of CESM examination with menstrual cycle was reported only in 18/84 studies (21%). In 10/18 (56%) articles, it was mentioned but not applied; in 6/18 (33%), it was applied with a feasibility window between the 5th and 14th day of menstrual cycle; in 2/18 (11%), CESM was synchronously performed with MRI in different phases of menstrual cycle to evaluate and compare background parenchymal enhancement.

### Technical features and parameters

In 70 out of 84 studies (83%), different systems from General Electric Healthcare (Chicago, IL, USA) were used, all with a prototype or a commercial release of the SenoBright upgrade which is required to perform dual-energy contrast-enhanced imaging. Twelve out of 84 articles (14%) reported the adoption of Selenia Dimensions Mammography Unit (Hologic Inc., Marlborough, MA, USA), while the remaining 2/84 (3%) studies were conducted with a Siemens Healthineers (Erlangen, Germany) Mammography System (Mammomat or Mammomat Inspiration).

The type of ICA used was not reported in five articles [15, 24, 64, 66, 75], while in the remaining 79 studies (94%), for a total of 13465 patients (96%), six different molecules were used: Iohexol was the most frequently employed, being used in 42/79 studies (53%) for a total of 5049/13465 patients (37%), followed by Iopromide (18/79 studies, 23%; 2798/13465 patients, 21%), while Iobitridol, Iomeprol, Iopamidol and Ioversol were administered in the remaining studies (19/79 studies, 24%; 5618/13465 patients, 42%). Iohexol was utilised at a concentration of 350 mg iodine/mL (25/42 studies, 60%; 3330/5049 patients, 66%) or 300 mg iodine/mL (17/42 studies, 40%; 1719/5049 patients, 34%). Iopromide was also administered at two different concentrations: 370 mg iodine/mL (10/18 studies, 56%; 1032/2798 patients, 37%) and 300 mg iodine/mL (8/18 studies, 44%; 1766/2798 patients, 63%).

Of the 69 studies including a specification of the contrast injection modality, 59 (85%) utilised an automated power injector (10584/11725 patients, 90%) while manual contrast injection was carried out in the remaining 10 (15%) [7, 9, 17, 25, 28, 51, 57, 73, 95, 99] for a total of 1141/11725 patients (10%).

Contrast agent dose, detailed in 77 studies, was fixed at 1.5 mL/kg in 72 (93%) of them for a total of 13559/13687 (99%) patients. Contrast agent flow rate, reported in 76/84 studies (90%), was most frequently fixed at 3 mL/s (65/76 studies, 86%); the 11 remaining articles detailed a flow rate ranging from 2 to 5 mL/s. Thirty-five out of 84 (42%) articles for a total 8734/14012 patients (62%) also mentioned the use of additional post-contrast saline flush or “bolus chaser,” 19 of them (54%, for a total 4477/8734 patients, 51%) likewise detailing a saline amount ranging from 10 to 30 mL.

Of 69 studies detailing the tube voltage of both low- and high-energy acquisitions, all but one (99%) acquired low-energy images between 26 and 33.2 kVp, which is the peak kilovoltage threshold of iodine, while all 69 acquired high-energy images well above this threshold, i.e. between 44 and 50 kVp. The anode/filter combination was reported by 42/84 studies. Exposure parameters were unambiguously reported only in one study [10], whereas in 5 early studies [7, 8, 32, 59, 85], they were manually adjusted according to breast thickness and density; thirty-five other studies declared an automatic regulation of these parameters performed by the mammography unit.

Regarding radiation dose, data were scarcer: even though 45/84 articles (54%) mentioned this aspect, 17/45 (31%) did it without exhibiting original information but reporting observations from previous studies, therefore restricting the number of studies with new data to 28/84 (33%). Of these 28 studies, 19 (68%) provided an average glandular dose (AGD), 3 (16%) of them calculating it per-patient and ranging 1.5–6.9 mGy [8, 9, 58], 5/19 (26%) calculating it per-breast ranging 2.19–7.15 mGy and the remaining 11 (58%) reporting a per-view AGD ranging from 0.43 [61] to 2.65 mGy [101]. A comparison with DM was mentioned in 17 studies: only 1 (6%) documented a dose reduction (− 2%) for CESM compared to DM [32], while other 16 (94%) reported an increase in AGD ranging between 6.2% [85] and 100% [77]. However, it is worth to notice that 3 studies specifically contrived to assess CESM radiation doses reported an AGD increase of 42% [56], 78% [82] and 80% [60].

### Acquisition protocols

Studies reporting the time interval between contrast injection and the first image acquisition were 78 out of 84 (93%), for a total 13244/14012 patients (95%) and 65 (83%) of them (12278/13244 patients, 93%) had it fixed at 120 s.

Sixty-six out of 84 articles (79%, 11900/14012 patients, 85%) gave an indication of the acquisition time after contrast injection: in 12/66 (18%, 1381/11900 patients, 11.6%), the exam was completed in less than 5 min; in 52/66 (80%, for total of 10485/11900 patients, 88.1%) between 5 and 10 min, while in 1/66 (2%, 34/11900 patients, 0.3%) the duration exceeded 10 min.

The outline of the image acquisition sequence remains more variable. Ten out of 84 studies (12%), accounting for 2734 patients (19%) did not clearly describe it and did not provide a reference to other protocols, while 3/84 (4%, 103/14012 patients, 1%) employed a curtailed and side-insensitive acquisition sequence. Adherence to standard but unspecified digital mammography protocols was declared by 29/84 (34%) studies, for total 3741/14012 patients (27%). The other half of the articles analysed (42/84, accounting for 7434/14012 patients, 53%) unequivocally detailed an acquisition sequence. Of these 42 studies, 14 (34%, 2048/7434 patients, 28%) adopted a projection order that was conventionally agreed upon, while the other 28 (66%, accounting for 5386/7434 patients, 72%) based their acquisition sequence on the presence of previous suspect or clearly pathologic findings.

**Fig. 3:**
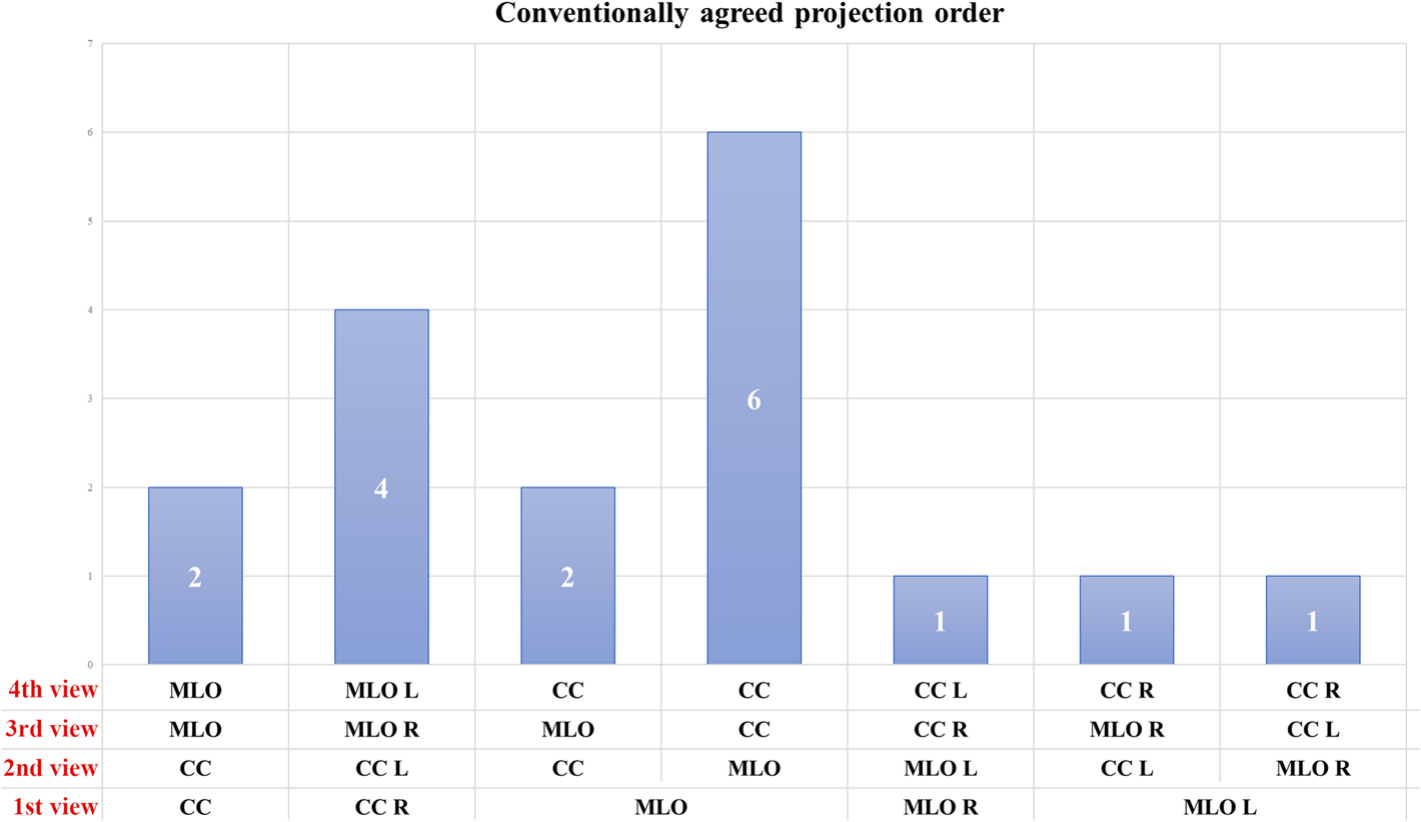
Graphical summary of conventionally agreed view acquisition orders for contrast-enhanced spectral mammography: *CC* craniocaudal view, *MLO* mediolateral oblique view, *L* left, *R* right

Eighty-four articles came from 38 different research groups. Subgroup analysis according to research groups showed that 17 acquisition sequences based on a conventionally agreed projection order were executed in 15 research groups. As described in Fig. 3, the most common sequence description, reported by 6/17 (35%) institutions, was MLO - MLO - CC - CC (in order of acquisition), without any further indication about the first side to be examined (right or left or side with/without suspicious lesion or already diagnosed cancer). The second most common sequence (4/17, 24%) was CC - CC - MLO - MLO with the first projection standardised on the right side (independently of pathology or with suspected pathology).

**Fig. 4:**
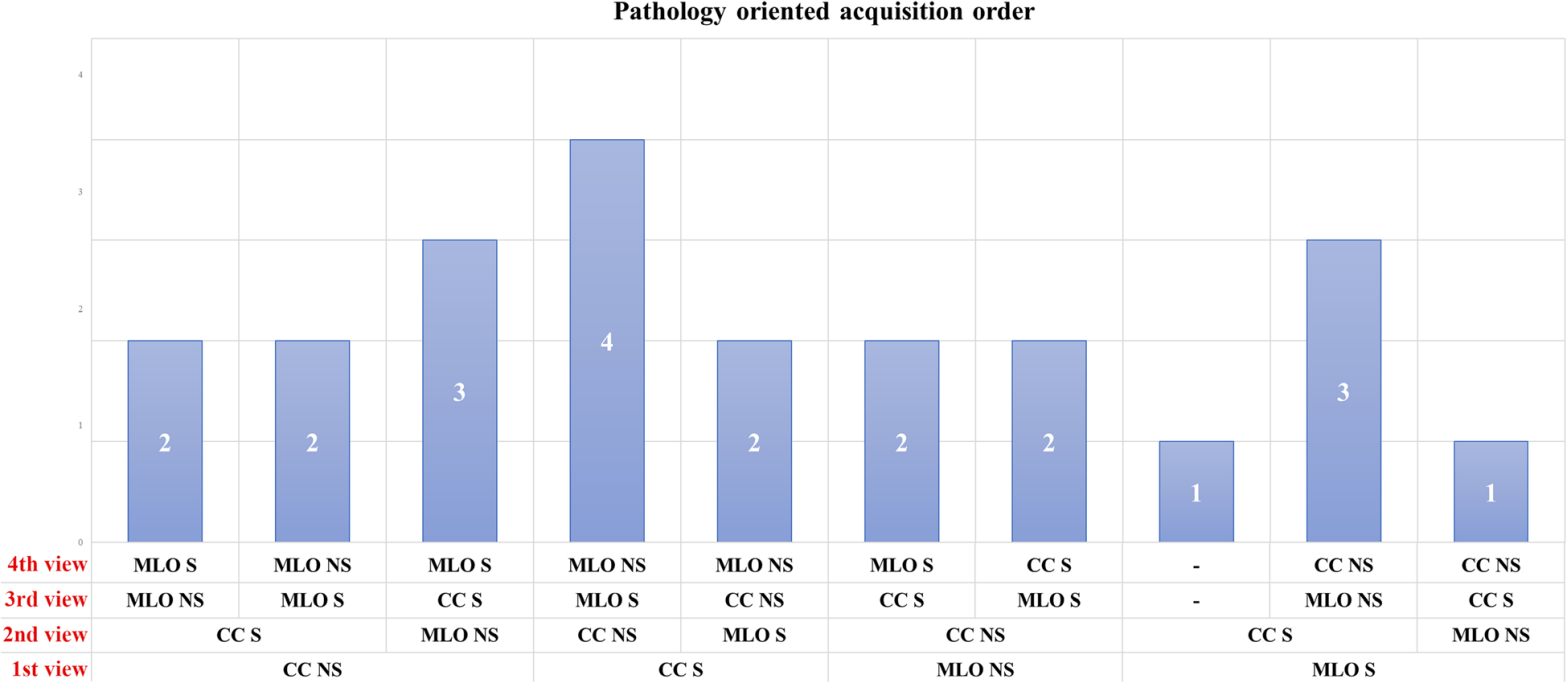
Graphical summary of pathology-oriented view acquisition orders for contrast-enhanced spectral mammography: *CC* craniocaudal view, *MLO* mediolateral oblique view, *S* suspicious breast, *NS* not suspicious breast

Among the 22 acquisition sequences (coming from 20 institutions) centred on the presence of previous suspect or clearly pathologic findings, we found substantial variability between different orders of acquisition, as shown in Fig. 4. However, the most common sequence, adopted by 4/22 (19%) research groups, was 1) CC, suspected side; 2) CC, non-suspected side; 3) MLO, suspected side; and 4) MLO, non-suspected side.

### Contrast agent adverse reaction rate meta-analysis

Regarding side effects from ICA administration, 48/84 studies (57%) declared a preventive anamnestic screening for previous adverse reactions or general contraindications to ICA administration. Pre-examination tests of renal function was mentioned in 39/84 studies (46%). Of note, 14/84 studies (29%) reported 30 adverse reactions out of 14012 patients, of which 26/30 (87%) were mild reactions limited to pruritus, hives, “scratchy throat” or other minor skin flushing that resolved promptly even when antihistamines or corticosteroids were not administered. In 3/30 (10%) cases [54, 58, 87], side effects were of moderate importance with nausea and vomiting, widespread urticaria resolved only after antihistamines and corticosteroids per os, and dyspnea that equally responded to oral antihistamine administration. Only 1/30 (3%) severe adverse reaction, requiring “intensive care” but resolved after short time, occurred in 14012 patients (0.007%) [61].

**Fig. 5:**
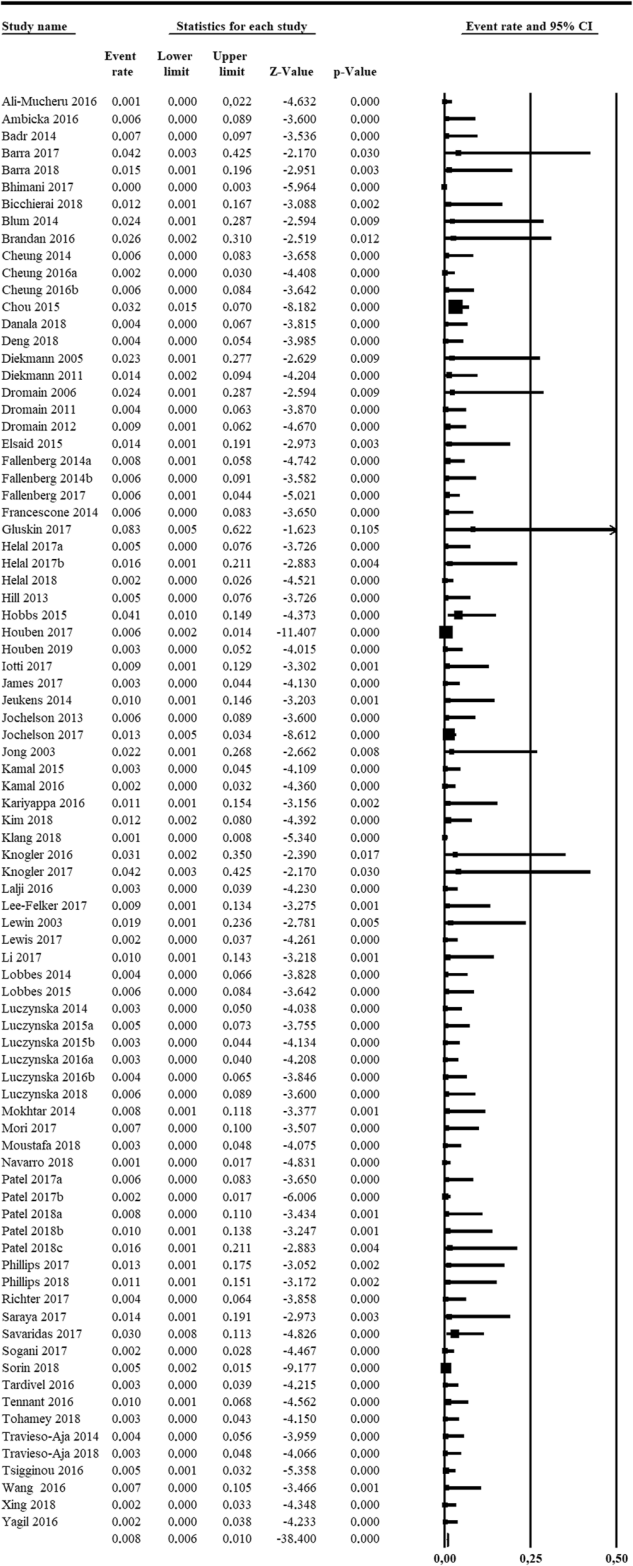
Forest plot of the 84 analysed articles on contrast-enhanced spectral mammography. No heterogeneity was found among studies (*I*^2^ = 0%). The last row shows the pooled rate for adverse reactions arising from iodinated contrast agent administration, calculated using the fixed-effect model

Therefore, the number of adverse reactions related to ICA administration ranged from 0, reported by 70 (88%) studies, to a maximum of 6 adverse reactions [14] with a total of 30 adverse reactions, showing no heterogeneity (*Q* = 64, degree of freedom 83, *τ* = 2.0972, *I*^2^ = 0%, *p* = 0.931). As shown in the forest plot of Fig. 5, using fixed-effect model, the pooled rate of adverse reactions across studies was 0.82%, with 0.64% and 1.05% as 95% CI.

**Fig. 6:**
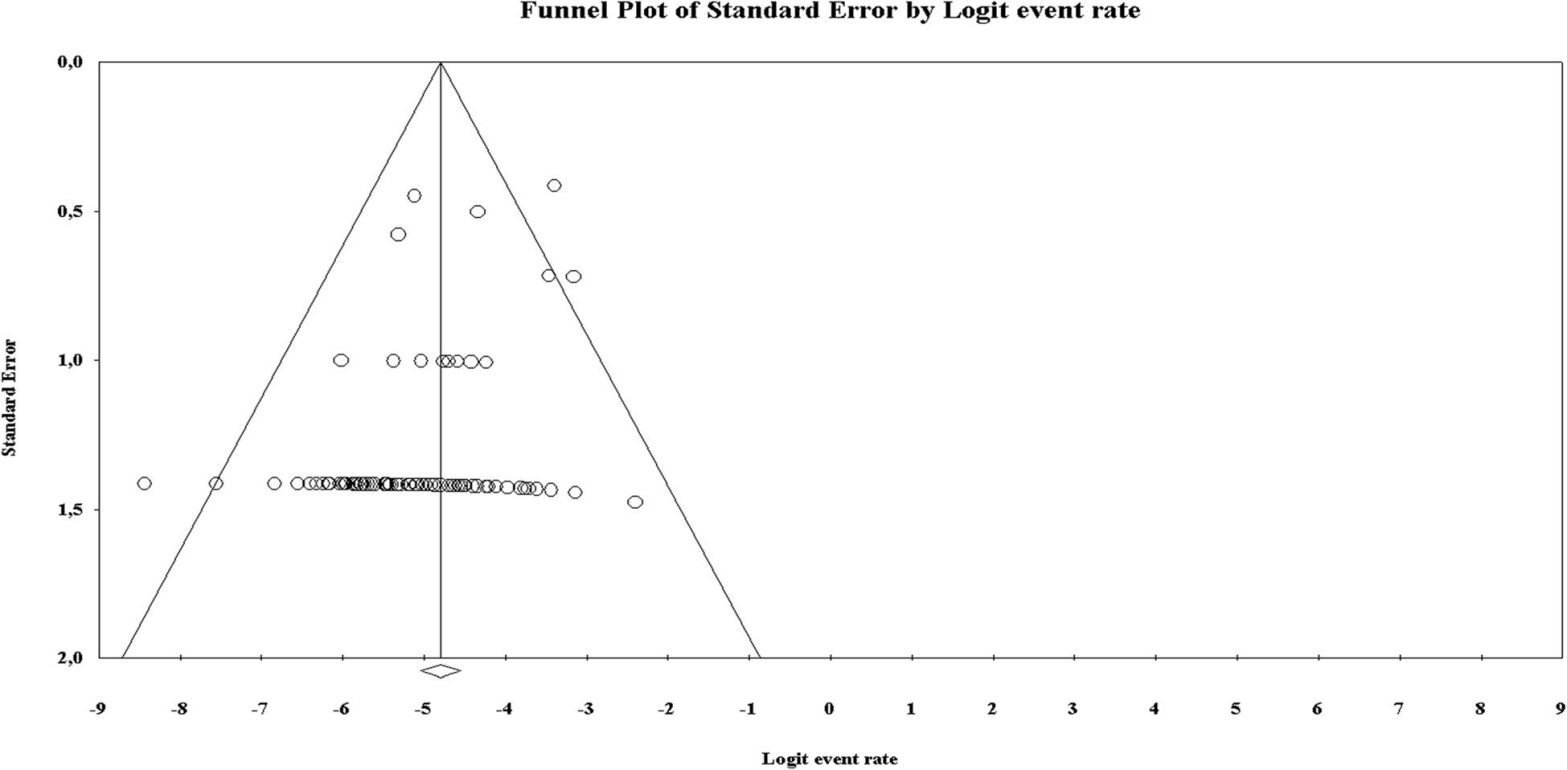
Funnel plot showing risk of publication bias in articles on contrast-enhanced spectral mammography, confirmed by the Egger test (*p* < 0.001)

Visually inspecting the funnel plot in Fig. 6, risk of publication bias was found, as confirmed by the Egger test (*p* = 0.00028).

## Discussion

**Fig. 7:**
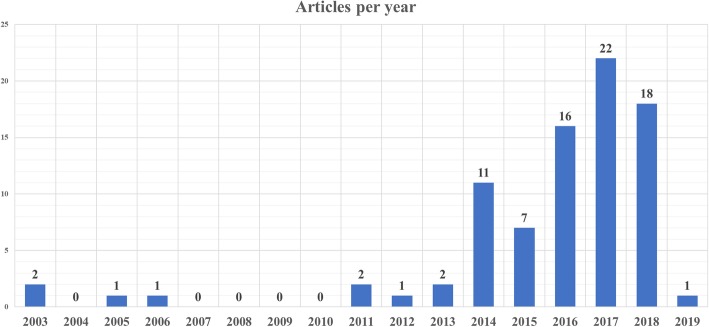
Graphic showing the number of articles published per year regarding contrast-enhanced spectral mammography

Our systematic review included 84 articles, accounting for 14012 patients, reporting the use of CESM in various settings. The sheer number of studies and, as depicted in Fig. 7, their increase in the last 3 years (27 studies between 2003 and December 2015, 57 from January 2016 to January 2019) points out a considerable interest in this emerging breast imaging modality.

A number of narrative reviews [6, 42, 102–106] favourably outlined CESM future perspectives in several clinical settings (e.g. recall work-up, pre-operative staging, and monitoring the effect of neoadjuvant therapy) as a potential alternative to MRI.

In the first phase of CESM development, some non-fixed parameters regarding contrast agent administration (i.e. contrast agent molecule, concentration, dose, flow rate, and injection modality) and some acquisition features (i.e. time between contrast injection and first acquisition, kVp ranges for low- and high-energy acquisitions) gained an international agreement. However, in the framework of comprehensive optimisation and standardisation of CESM, large-scale studies are undoubtedly needed to address the knowledge gap concerning the choice of technical parameters.

Our data show a consensus among studies (93%) on the choice of 1.5 mL/kg contrast dose administered with a 3 mL/s flow rate (74%) and a less extensive agreement on the use of Iohexol (53% of all studies) at a concentration of 350 mg iodine/mL (30% of all studies). However, these parameters have probably been empirically adopted from CT protocols, as the first investigators plainly stated [7], without any other particular explication or justification. No dose-finding studies have been published yet.

Similarly, the common use of a power contrast injector (87% of all studies, with the remaining 13% coming from a single research group) is assumed from CT and MRI protocols in which it has been demonstrated to be effective in obtaining a stable contrast inflow and bolus shape [107–109]. Moreover, the use of a power injector allows for the administration of a bolus chaser, reported only in 42% of all articles, a technical refinement that has shown good results in CT [110, 111].

Two other points need to be mentioned. The first one is the correlation between menstrual cycle phase and background parenchymal enhancement, explored in a few studies [10, 75, 80] and/or fluctuations of lesion contrast uptake. Secondly, since CESM is based on a dual X-ray exposure, of which the low-energy one has been demonstrated to be equal to standard DM [66], an increase in radiation dose is expected. However, while preliminary studies estimated a negligible [7] or curtailed AGD increase, studies specifically devised to ascertain CESM effective AGD found a substantial AGD increment ranging 42–80% [56, 60, 82]. While CESM AGDs remain under the threshold stated by European guidelines for screening mammography [112], further studies are needed to investigate CESM AGD [56, 82].

Furthermore, we remark the absence of standardised protocols. This methodological void, especially regarding the acquisition workflow, represents a threat to reproducibility and comparison of imaging results. While 98% of all studies reporting the total examination time completed the examination before 10 min from contrast administration, and while some studies presented evidence on the irrelevance of the acquisition order [55, 64], there are no studies comparing different approaches.

The pooled rate of adverse reactions to ICA administration was 0.82% (0.64–1.05% 95% CI) with a total of 30 adverse reactions in 14012 patients, a rate similar to that reported for CT 0.6% [113] in 84928 adult patients or 0.7% [114] in 29508 patients (given Iopromide, which is also used for CESM). Particularly, considering only severe adverse reactions in CT, Wang et al. [113] reported 11/84928 (0.0129%) reactions, as well as Mortelé et al. [114] 4/29508 (0.0135%). These rates seem to be higher than that found in our meta-analysis 1/14012 (0.007%), a comparison to consider with caution due to the nature of rare events such as severe reactions to ICA. One aspect to consider is the different profile of patients undergoing CESM compared to those requiring contrast-enhanced CT, the former being that of basically “healthy” subjects, the latter implying the possibility of relevant disease, including also serious emergency conditions.

This review has limitations. Patient data are probably shared and duplicate among some studies from the same research group. This has been shown to negatively impact on review quality [115, 116] and could only be prevented via individual patient data sharing [117]. However, for technical aspects of this systematic review, our choice to evaluate study groups rather than single articles should have mitigated this bias. Conversely, our pooled rate of adverse reactions could be underestimated.

In conclusion, our review shows that CESM is unevenly performed across different centres, in terms of contrast agent type and concentration and order of view acquisition. However, most research groups performed CESM using a contrast dose of 1.5 mL/kg, factory-set kVp ranges for low- and high-energy acquisitions, beginning image acquisition after 120 s from contrast agent injection and completing the examination within 10 min. Further studies are needed to investigate the role of background parenchymal enhancement and to harvest data that can firmly back up subsequent technical guidelines and consensus statements for standardised CESM protocols.

## Data Availability

The datasets used and analysed during the current study are available from the corresponding author on reasonable request.

## Abbreviations

AGD: Average glandular dose
CC: Craniocaudal
CESM: Contrast-enhanced spectral mammography
CI: Confidence interval
CT: Computed tomography
DM: Digital mammography
ICA: Iodinated contrast agent
kVp: Peak kilovoltage
MLO: Mediolateral oblique
MRI: Magnetic resonance imaging
PRISMA: Preferred Reporting Items for Systematic Reviews and Meta-Analyses
